# Protective Role of Vanillic Acid against Diethylnitrosamine- and 1,2-Dimethylhydrazine-Induced Hepatocarcinogenesis in Rats

**DOI:** 10.3390/molecules26092718

**Published:** 2021-05-05

**Authors:** Charatda Punvittayagul, Arpamas Chariyakornkul, Kanokwan Jarukamjorn, Rawiwan Wongpoomchai

**Affiliations:** 1 Research Affairs, Faculty of Veterinary Medicine, Chiang Mai University, Chiang Mai 50100, Thailand; charatda.pun@cmu.ac.th; 2 Department of Biochemistry, Faculty of Medicine, Chiang Mai University, Chiang Mai 50200, Thailand; arpamas.c@gmail.com; 3 Research Group for Pharmaceutical Activities of Natural Products Using Pharmaceutical Biotechnology, Faculty of Pharmaceutical Sciences, Khon Kaen University, Khon Kaen 40002, Thailand; kanok_ja@kku.ac.th

**Keywords:** 1,2-dimethylhydrazine, aberrant crypt foci, cancer chemoprevention, diethylnitrosamine, GST-P positive foci, vanillic acid

## Abstract

This study aimed to evaluate the cancer chemopreventive activity of vanillic acid (VA) in diethylnitrosamine- and 1,2-dimethylhydrazine-induced liver and colon carcinogenesis in rats. VA did not induce the formation of hepatic glutathione *S*-transferase placental form (GST-P) positive foci and colonic aberrant crypt foci, demonstrating no carcinogenic activity. VA (75 mg kg^−1^ body weight) could significantly reduce the number and areas of hepatic GST-P positive foci when administered before carcinogen injections, but no such effect was seen when it was administered after carcinogen injection. No protection was seen in the colon when VA was treated before or after carcinogen injection. Immunohistochemical studies demonstrated the decreased expression of proliferating cell nuclear antigen and the induction of apoptosis. Mechanistic studies showed that VA significantly induced the expression of *GSTA-5* and *Nrf-2* genes, which are associated with the detoxification system. Likewise, the antiproliferative effect was noticed by the reduction of *Cyclin D1* expression. The apoptotic activity may be due to the upregulation of *Caspase-3* and *Bad* levels and downregulation of the *Bcl-2* level. These data suggest that VA exhibited significant protection against diethylnitrosamine- and 1,2-dimethylhydrazine-induced hepatocarcinogenesis, which might be related to the induction of the detoxifying enzyme, the reduction of proliferation and the induction of apoptosis.

## 1. Introduction

Today, the trend for food as an attractive source of therapeutic candidates for various diseases is increasing. Numerous reports have supported the use of foods as drugs in the prevention and treatment of diseases, including cancer. It is well-known that the mechanism of carcinogenesis involves multiple factors, such as oxidative stress, inflammation and tumor suppressor gene or oncogene mutations [1]. Previous studies have reported that several plant-derived compounds in food are currently successfully employed in cancer prevention and treatment. For example, curcumin and resveratrol were used in a clinical trial for colorectal cancer treatment [2]. Pre-clinical studies have demonstrated that tea polyphenol and soy isoflavone presented chemopreventive activity against prostate cancer [3]. Lycopene from tomatoes decreased both DNA damage and the tumor size of prostate cancer in human studies [4]. Although many studies have reported the anti-cancer activity of various natural products, the search for more natural anticarcinogenic agents is still necessary.

**Figure 1:**
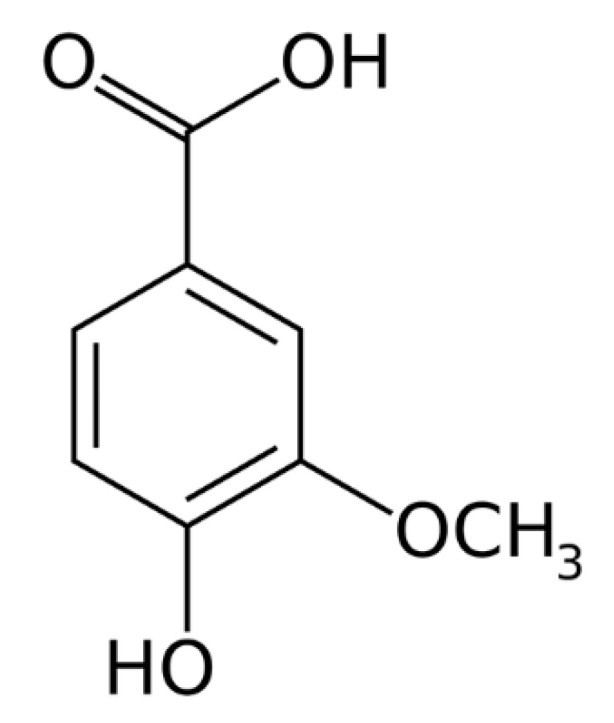
The chemical structure of vanillic acid.

Vanillic acid (VA) (4-hydroxy-3-methoxybenzoic acid, Figure 1) is an oxidized form of vanillin. It is a phenolic compound found in various plants such as rice, rye and almond skins [5,6,7]. Numerous studies have presented its biological activities, such as antioxidant, anti-inflammation and antimutagenicity activities. VA has presented in vitro antioxidant activity and has also been shown to be protective against H_2_O_2_- and UV-induced DNA damage in pBR322 plasmid DNA [8]. It has also reduced insulin resistance and improved glucose homeostasis in rats made diabetic hypertensive by a high fat diet [9]. In addition, VA has exhibited anti-inflammatory effects against LSP-induced inflammation in mouse peritoneal macrophages [10] and isoproterenol-induced cardiotoxicity in rats [11]. It has shown a hepatoprotective effect against concanavalin A-induced liver injury in mice [12]. Furthermore, our previous study found that VA displayed antimutagenicity in a *Salmonella* mutation assay and a rat liver micronucleus test [13]. However, the anti-cancer effect of VA on diethylnitrosamine- and 1,2-dimethylhydrazine-induced liver and colon carcinogenesis in rats and its inhibitory mechanisms have never been conclusively shown. 

The present study aimed to determine the anticarcinogenic effect of VA against diethylnitrosamine- and 1,2-dimethylhydrazine-induced liver and colon carcinogenesis in rats. In addition, the inhibitory mechanisms involving cell proliferation and apoptosis were also determined. 

## 2. Results

### 2.1. Effect of VA on General Observations, Relative Organ Weights and Serum Aspartate Aminotransferase (AST) and Alanine Aminotransferase (ALT) in Diethylnitrosamine-(DEN) and 1,2-Dimethylhydrazine-(DMH) Initiated Rats

There was no difference in food or water consumption among the various experimental groups (data not shown). Treatment with DEN and DMH significantly decreased the final body weight when compared with the negative control. However, treatment with VA at concentrations of 0.75 or 75 mg kg^−1^ body weight (BW) did not alter the final body weight when compared with the positive control group. In addition, treatment with VA at a concentration of 75 mg kg^−1^ BW alone maintained the body weights of rats when compared with the negative control group. There was no significant difference in relative liver, spleen or kidney weights of rats in each group. The levels of serum AST and ALT and the biochemical markers of hepatic cell damage were significantly increased in the DEN- and DMH-treated group, indicating that DEN and DMH induced rat liver damage. However, treatment with 0.75 and 75 mg kg^−1^ BW of VA did not alter the level of serum AST and ALT in the DEN- and DMH-treated group. The results are summarized in Table 1. These results showed that VA at the concentrations used in this study had no adverse effect on rats. 

### 2.2. Effect of VA on Preneoplastic Lesion Formation in Livers and Colons of DEN- and DMH-Initiated Rats 

This study aimed to determine the cancer chemopreventive effect of VA on liver and colon carcinogenesis induced by DEN and DMH. Hepatic glutathione *S*-transferase placental form (GST-P) positive foci using immunohistochemistry and colonic aberrant crypt foci (ACF) using methylene blue staining were used as the end-point markers of carcinogenesis, as we have reported previously [14]. We found that treatment with VA at a concentration of 75 mg kg^−1^ BW did not induce the formation of hepatic GST-P positive foci and colonic ACF. These findings suggest that VA did not present carcinogenicity in rats. Interestingly, treatment with VA at a concentration of 75 mg kg^−1^ BW prior to DEN and DMH injection significantly decreased the number and areas of the GST-P positive foci in rat livers, while a low dose of VA did not inhibit carcinogenicity. On the other hand, treatment with 0.75 and 75 mg kg^−1^ BW of VA after a DEN and DMH injection did not affect the number of the GST-P positive foci. In addition, treatment with VA either prior to or after a DEN and DMH injection did not show any inhibitory effect on colonic ACF formation. The results are shown in Table 2. These findings indicate that VA at a concentration of 75 mg kg^−1^ BW presented a chemopreventive effect on DEN- and DMH-induced carcinogenesis in rats. 

### 2.3. Effect of VA on Cell Proliferation and Apoptosis

**Figure 2:**
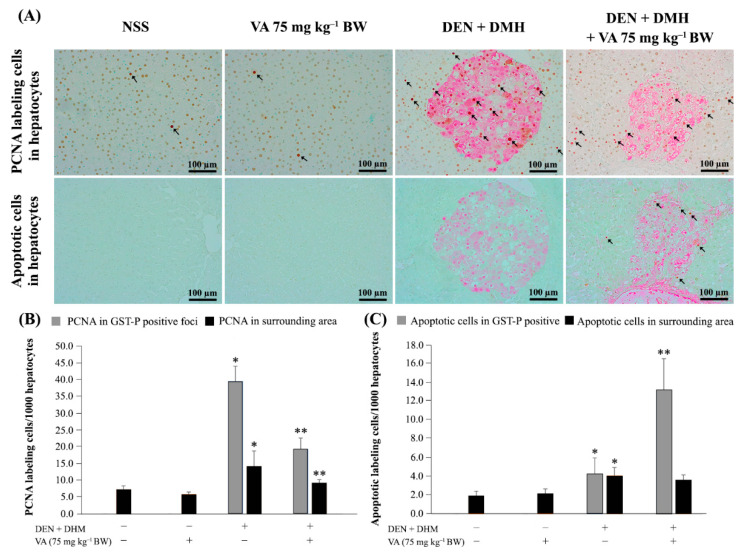
The effect of vanillic acid on cell proliferation and apoptosis. (**A**) Double-staining immunohistochemistry of PCNA positive cells and apoptotic cells in hepatocytes (20×). Arrowheads, stained hepatocytes. (**B**) The number of PCNA labelling cells/1000 hepatocytes (**C**) The number of apoptotic cells/1000 hepatocytes. The values are expressed as mean ± SD. * Significantly different from negative control group, *p* < 0.05. ** Significantly different from positive control group, *p* < 0.05. GST-P: glutathione *S*-transferase placental form, PCNA: proliferating cell nuclear antigen, DEN: diethylnitrosamine, DMH: 1,2-dimethylhydrazine, NSS: normal saline solution, VA: vanillic acid.

Following the result that 75 mg kg^−1^ BW of VA inhibited rat hepatocarcinogenesis, the inhibitory mechanisms involving cell proliferation and apoptosis in rat livers were determined by double-staining immunohistochemistry. The differences between proliferative and apoptotic cells of the GST-P positive foci and the surrounding areas between the DEN- and DMH-treated group and the negative control group were statistically significant. The treatment with 75 mg kg^−1^ BW of VA significantly decreased the number of proliferating cell nuclear antigens (PCNAs) in both the GST-P positive foci and the surrounding areas. In addition, VA also significantly enhanced cell apoptosis in the GST-P positive foci, but it had no effect on the surrounding tissues. The PCNA and apoptotic labeling of cells in the GST-P positive foci and the surrounding areas is shown in Figure 2. These findings indicate that VA inhibited rat hepatocarcinogenesis by the reduction of cell proliferation and the induction of cell apoptosis.

### 2.4. Effect of VA on the mRNA Levels of Genes Involved in the Early Stages of Hepatocarcinogenesis 

**Figure 3:**
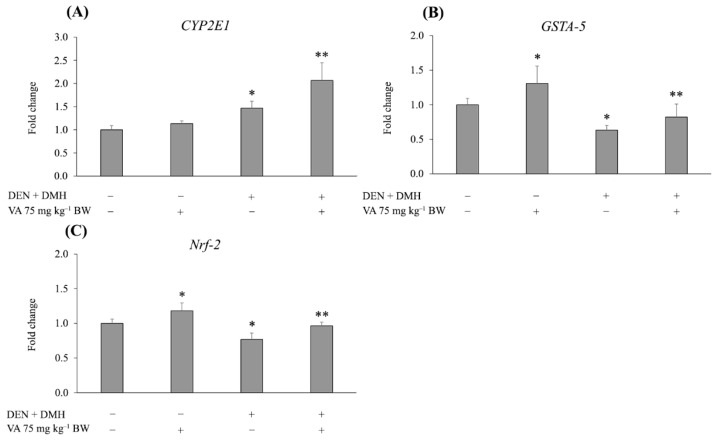
The effect of VA on the expression of genes of some Phase I and Phase II enzymes. (**A**) *CYP2E1*: cytochrome P450 2E1. (**B**) *GSTA-5*: glutathione *S*-transferase A5. (**C**) *Nrf-2*: nuclear factor erythroid 2-related factor 2. Values are expressed as mean ± SD. * Significantly different from negative control group, *p* < 0.05. ** Significantly different from positive control group, *p* < 0.05. DEN: diethylnitrosamine, DMH: 1,2-dimethylhydrazine, NSS: normal saline solution, VA: vanillic acid.

The expression of genes that are associated with the development of liver cancer, including xenobiotic metabolizing enzymes, cell proliferation and apoptosis, was determined by a real-time polymerase chain reaction. The result showed that treatment with DEN and DMH significantly increased the mRNA levels of *CYP2E1*, a major Phase I enzyme for DEN and DMH biotransformation, when compared with the negative control group. Interestingly, the treatment with VA at a concentration of 75 mg kg^−1^ BW significantly enhanced the mRNA levels of *CYP2E1* in DEN- and DMH-initiated rats when compared with the positive control group. The expression of *GSTA-5*, a detoxifying enzyme, was significantly upregulated in the VA alone group, while DEN and DMH treatment significantly downregulated *GSTA-5* expression. However, treatment with VA significantly induced the expression of *GSTA-5* in the DEN- and DMH-treated group. These results were associated with the mRNA level of *Nrf-2*, the transcription factor of the GST enzyme. The results are shown in Figure 3A–C. These findings demonstrate that VA inhibited DEN- and DMH-induced hepatocarcinogenesis by the induction of a Phase II detoxifying enzyme (GST) through the activation of the Nrf-2 signaling pathway. 

**Figure 4:**
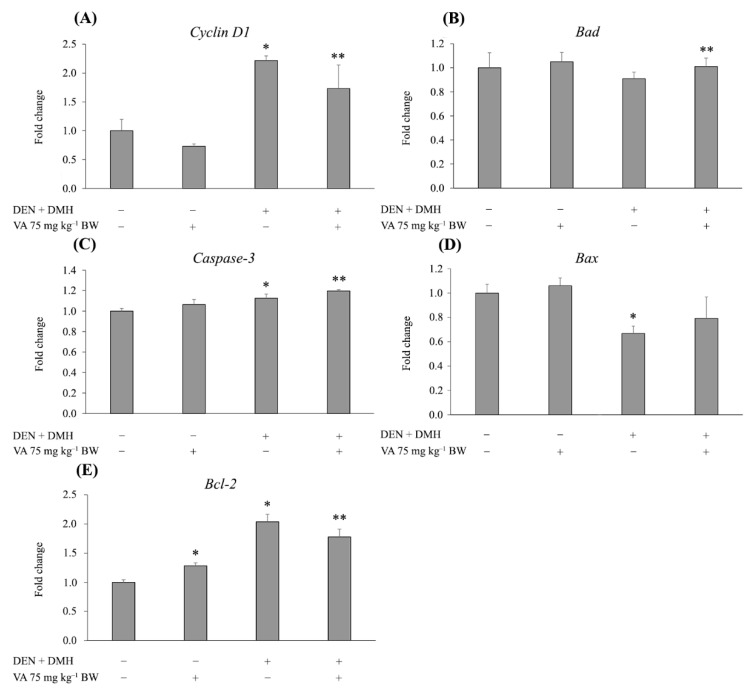
The effect of VA on the expression of genes related to cell proliferation and apoptosis. (**A**) Cyclin D1. (**B**) Bad. (**C**) Caspase-3. (**D**) Bax. (**E**) Bcl-2. Values are expressed as mean ± SD. * Significantly different from negative control group, *p* < 0.05. ** Significantly different from positive control group, *p* < 0.05. DEN: diethylnitrosamine, DMH: 1,2-dimethylhydrazine, NSS: normal saline solution, VA: vanillic acid.

The expression of genes that are associated with cell proliferation and apoptosis was determined in this study. The results showed that DEN and DMH significantly increased the expression of *Cyclin D1*, a key regulator of the G1/S phase transition, when compared with the negative control group. On the other hand, treatment with VA significantly decreased the expression of *Cyclin D1* in DEN- and DMH-initiated rats (Figure 4A). Additionally, it significantly enhanced the expression of the pro-apoptotic gene, *Bad*. A similar trend was found in *Caspase-3* expression. However, VA did not significantly affect the *Bax* expression in the livers of DEN- and DMH-initiated rats (Figure 4B–D). Moreover, VA significantly downregulated the expression of *Bcl-2*, an anti-apoptotic gene, in DEN- and DMH-treated rats (Figure 4E). These findings demonstrate that VA inhibited cell proliferation through the reduction of cyclin D1. The induction of cell apoptosis was associated with a combination of both the upregulation of pro-apoptotic genes and the downregulation of anti-apoptotic genes.

## 3. Discussion

Numerous studies have shown that foods and medicinal plants are considered important sources of bioactive compounds that present health-promoting properties. Phytochemicals contained in plant foods have been shown to prevent various diseases, including cancers. Therefore, bioactive compounds with demonstrated anti-cancer properties have been considered as an alternative therapeutic approach. In the present study, we investigated the anticarcinogenic effect of VA, a phenolic compound contained in various foods such as rice, rye and almond skins. Our previous study showed that VA presented antimutagenicity in a *Salmonella* mutation assay and anticlastogenic activity in rat livers [13]. The present study found that VA exhibited chemopreventive potential on DEN- and DMH-induced hepatocarcinogenesis in rats. 

Recently, we reported that treatment with DEN and DMH significantly increased the levels of AST and ALT, indicators of hepatic injury, when compared with the normal group [14]. Similar findings were observed in this study. However, treatment with VA either before or after carcinogen injections did not affect the levels of AST and ALT. These results showed that VA did not improve hepatotoxicity caused by DEN and DMH treatment. Additionally, we found that treatment with 75 mg kg^−1^ BW of VA before the DEN and DMH injections significantly reduced the areas and number of hepatic GST-P positive foci, compared with the positive control group. Our previous study found that VA could inhibit the formation of rat liver micronucleus induced by AFB_1_ [13]. These findings showed that VA can specifically inhibit hepatocarcinogenesis induced by chemical carcinogens. A previous report demonstrated that free metabolites and a VA conjugated form are mainly eliminated in urine [15]. This report could support our study that VA, which acts as an anti-cancer agent, might be absorbed and transferred to the liver. However, its conjugated form is excreted in urine, resulting in no anti-cancer activity in the colon. 

It is known that DEN and DMH are primarily metabolized by cytochrome P450 2E1 (CYP2E1) to electrophilic metabolites. The intermediates could further react with DNA to form DNA adducts, resulting in DNA mutation and cancer development [16,17]. In addition, CYP2E1-mediated oxidation also generates reactive oxygen that can cause oxidative stress and cell toxicity [18]. We found that treatment with DEN and DMH enhanced the expression of *CYP2E1*, which is related to hepatotoxicity. However, VA treatment, in combination with DEN and DMH, resulted in higher *CYP2E1* expression than in the positive controls. It is possible that VA could not inhibit hepatotoxicity in rats due to the induction of *CYP2E1*. Nevertheless, the reactive intermediates could detoxify Phase II enzymes such as glutathione *S*-transferase, which are regulated by nuclear factor erythroid 2 (NFE2)-related factor 2 (Nrf2) [19]. GST, in addition to performing the role of detoxification, also acts as an antioxidant enzyme. A previous report has demonstrated that the induction of GST activity is an adaptive mechanism to attenuate CYP2E1-derived oxidative stress [20]. In the present study, although VA increased *CYP2E1* mRNA levels in the DEN- and DMH-treated rats, VA was able to increase *GSTA5* gene expression through the induction of *Nrf-2* expression. These findings may indicate that VA inhibits the initiation stage of rat hepatocarcinogenesis by inducing the excretion of reactive intermediates via the Nrf-2 signaling pathway.

An imbalance between cell proliferation and apoptosis plays an important role in cancer development. Cell proliferation is essential for the expansion of initiated cells, while apoptosis plays a critical role in growth control that is activated by DNA damage and uncontrolled proliferation [21,22]. Therefore, the induction of tumor cell death by apoptosis is a major goal of cancer treatment. In this study, VA showed a significant decrease of the PCNA positive cells in both the GST-P positive foci and the surrounding areas. The administration of VA significantly decreased the level of *Cyclin D1* gene expression when compared with the positive control group. It is well known that cyclin D1 is a protein that plays an important role in the G1/S phase progression of the cell cycle [23]. The evidence presented here strongly supports the idea that VA suppresses cell proliferation by the reduction of *Cyclin D1*, resulting in cell cycle arrest in the G1 phase, thus inhibiting cell proliferation. The balance between pro-apoptotic and anti-apoptotic proteins is recognized as an important factor for the regulation of cell death [24]. Anti-apoptotic proteins such as Bcl-2, Bcl-xl and Mcl-1 protect the cell from apoptotic stimuli, whereas pro-apoptotic proteins such as Bad, Bid, Bax and Bak are activated by cellular stress and DNA damage [25]. In the present study, VA significantly upregulated the expression of the pro-apoptotic gene *Bad*, and downregulated the anti-apoptotic gene *Bcl-2*, when compared with the DEN- and DMH-treated group. Additionally, it also upregulated the expression of the *Caspase-3* gene. These findings provide additional evidence that VA could induce cell apoptosis by a combination of both the overexpression of pro-apoptotic genes and the underexpression of anti-apoptotic genes. 

In the current study, the effective dose of VA for presenting cancer chemopreventive activity was 75 mg kg^−1^ BW, which corresponds to a human-equivalent dose (HED) of 730 mg/day in a 60 kg human [26]. The body might receive VA from the consumption of vegetables and fruits containing either VA or its parent flavonoids, such as catechin, which can be metabolized by gut microflora [27]. Therefore, the regular consumption of a plant-based diet may provide an adequate VA content for cancer prevention.

## 4. Materials and Methods

### 4.1. Chemicals 

Diethylnitrosamine, 3,3′-diaminobenzidine tetrahydrochloride hydrate and VA were purchased from Sigma-Aldrich (St. Louis, MO, USA). 1,2-Dimethylhydrazine dihydrochloride was obtained from TCI (Tokyo, Japan). Methylene blue and an ApopTag^®^ Peroxidase In Situ Apoptosis Detection Kit were purchased from Merck (Darmstadt, Germany). Anti-rat glutathione *S*-transferase placental form was obtained from MBL (Nagoya, Japan). A Vectastrain ABC kit was obtained from Vector Laboratories, Inc. (Burlingame, CA, USA). An EnVision Doublestain system was purchased from Dako (Hamburg, Germany). All other chemicals were of analytical grade. 

### 4.2. Animals and Treatment Protocol

Male Wistar rats (4 weeks old, 90–110 g) from the National Laboratory Animal Center, Mahidol University, Salaya, Nakhon Pathom, Thailand, were housed and acclimated for one week before the start of the experiments. They were maintained with a 12 h light/dark cycle at a constant temperature and humidity. Each animal had free access to food and water. The experimental procedure was approved by The Animal Ethics Committee of Faculty of Medicine, Chiang Mai University, and was performed in accordance with the institutional guidelines (Approval No. 37/2559). 

**Figure 5:**
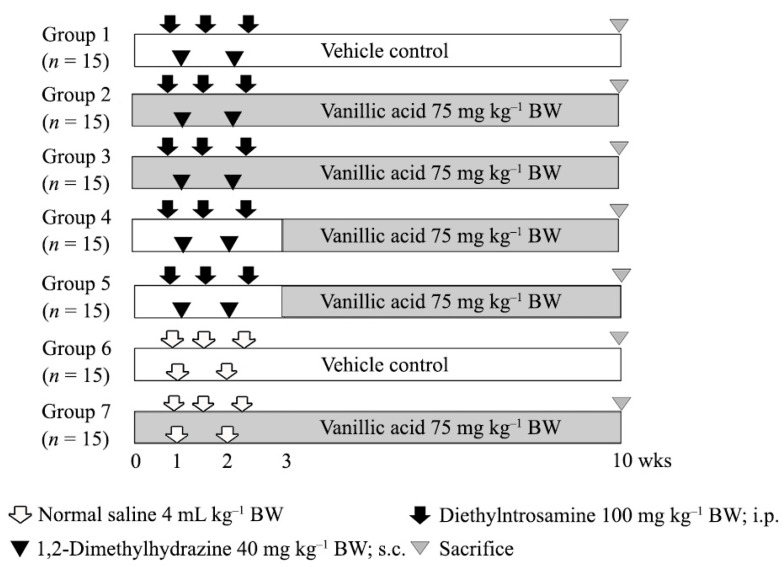
Experimental protocol.

The concentrations of VA were 0.75 and 75 mg kg^−1^ BW were calculated based on the VA content found in the rice husk used in our previous study [28]. Rats were randomly assigned to 7 groups. Groups 1–5 were intraperitoneally injected 3 times with diethylnitrosamine (DEN; Day 0, 4, 11) and subcutaneously injected 2 times with 1,2-dimethylhydrazine (DMH; Day 0, 7) to initiate liver and colon carcinogenesis, respectively. Group 1 was the positive control group. One week before the injection of carcinogens, groups 2 and 3 were treated with 0.75 or 75 mg kg^−1^ BW of VA via intragastric gavage feeding, respectively, to determine its protective effect. Groups 4 and 5 received 0.75 or 75 mg kg^−1^ BW of VA, respectively, after the carcinogen injection, to study its inhibitory effect. Group 6 was the negative control group, while group 7 received 75 mg kg^−1^ BW of VA to evaluate its carcinogenicity. All rats were sacrificed at week 10 of the experiment. The experimental protocol is shown in Figure 5. Body weight, water intake and food consumption were measured every week throughout the experiment. Blood samples were collected to analyze serum aspartate aminotransferase (AST) and alanine aminotransferase (ALT) levels. Liver and colon were removed and fixed in a 10% formaldehyde buffer for preneoplastic lesion measurement, using immunohistochemistry and methylene blue staining. The remaining portion of the liver was snap-frozen in liquid nitrogen and kept in −80 °C for molecular studies.

### 4.3. Determination of Serum AST and ALT Levels

Serum AST and ALT levels were measured by an Automatic biochemical analyzer using commercially available kits. 

### 4.4. Determination of Hepatic and Colonic Preneoplastic Lesions in Rats

**Figure 6:**
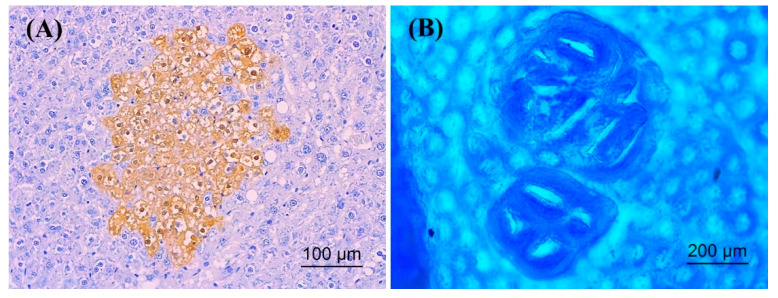
The preneoplastic lesions; (**A**) hepatic GST-P positive foci (20×), (**B**) colonic aberrant crypt foci (10×).

The avidin–biotin complex method was used to stain the hepatic GST-P positive foci (Figure 6A), according to the method of Thumvijit et al. [29]. The number and the areas of the GST-P positive foci that were greater than 0.2 mm in diameter were determined using the LAS Interactive Measurement program. 

The colonic ACF (Figure 6B) were determined by methylene blue staining. The colon was expanded by a 10% formaldehyde buffer and then stained with 0.2% methylene blue for a few minutes, according to Punvittayagul et al. [14]. The number of ACF and aberrant crypts was counted under a light microscope. 

### 4.5. Determination of PCNA and Apoptotic Hepatocytes by Double-Staining Immunohistochemistry

The double-staining procedures were performed using an EnVision Doublestain system from Dako (Hamburg, Germany). Liver sections were prepared for immunohistochemical staining with anti-PCNA antibody (1:2000 dilution) and anti-GST-P antibody (1:1000 dilution), and were performed according to the manufacturer’s instructions. The number of PCNA-positive cells was counted under a light microscope, both inside and in the areas surrounding the GST-P positive foci. The PCNA labeling cell was randomly examined by counting 2000 hepatocytes/rat. 

The apoptotic hepatocytes were determined by terminal deoxynucleotidyl transferase dUTP nick-end labeling (TUNEL) assay. Double immunostaining for TUNEL and GST-P was performed using an ApopTag^®^ Peroxidase In Situ Apoptosis Detection Kit and an EnVision Doublestain system, according to Thumvijit et al. [29]. The number of apoptotic cells was counted under a light microscope, in both the GST-P positive foci and the GST-P negative areas. The apoptotic labeling cell was determined by counting 2000 hepatocytes.

### 4.6. Gene Expression Analysis by Real-Time Polymerase Chain Reaction 

The total RNA was isolated from the rat livers using PureZOL™ RNA Isolation Reagent (Bio-Rad, Hercules, CA, USA), following the manufacturer’s instructions. cDNA synthesis was carried out using a High-Capacity cDNA Reverse Transcription Kit, (Applied biosystems™, Foster City, CA, USA), according to the supplier’s procedure. Quantitative real-time PCR was performed using specific primers (Integrated DNA Technologies, Inc., Singapore), as listed in Table 3. Real-time PCR was carried out using a SensiFast™ SYBR Lo-ROX Kit (Bioline, London, UK). PCR conditions consisted of initial denaturation at 95 °C for 1 min, 40 cycles of denaturation at 95 °C for 15 s, annealing at 56–60 °C for 15 s, and extension at 72 °C for 10 s. The expression of target genes was normalized using β-actin and the fold change in the expression of each gene was calculated by the 2^−ΔΔCT^ method. 

### 4.7. Statistical Analysis

Data are expressed as mean ± SD of each variable for each group. The significance of differences between groups was analyzed using one-way variance analysis, and least significant difference (LSD) was used to determine significantly different groups. *p*-Values less than 0.05 were significant.

## 5. Conclusions

The results of the present study conclusively demonstrate that VA exerts a cancer chemopreventive effect against DEN- and DMH-induced hepatocarcinogenesis. The anticarcinogenic effect of VA on the early stages of hepatocarcinogenesis involves the induction of the detoxifying enzyme GST via the Nrf-2 signaling pathway. VA decreases cell proliferation by reducing the expression of the *Cyclin D1* gene. Meanwhile, it also induces cell apoptosis by the induction of the expression of *Bad* and *Caspase-3* genes and the reduction of *Bcl*-2 gene expression. Thus, VA may be an attractive candidate as an anti-tumor drug for cancer treatment.

## Figures and Tables

**Table 1:** The effect of VA on relative organ weight and activities of serum AST and ALT in DEN- and DMH-initiated rats. Values expressed as mean ± SD.

Treatment		Week of Administration	Final Body Weight (g)	Relative Organ Weight (%)			AST (U/L)	ALT (U/L)
Carcinogen/NSS	VA (mg kg^−1^ BW)			Liver	Spleen	Kidney		
DEN + DMH	-	-	398.8 ± 22.8 *	3.35 ± 0.14	0.25 ± 0.03	0.57 ± 0.03	109.5 ± 25.7 *	64.8 ± 19.1 *
DEN + DMH	0.75	10	383.8 ± 19.8	3.26 ± 0.29	0.24 ± 0.02	0.55 ± 0.03	116.4 ± 29.0	61.6 ± 8.9
DEN + DMH	75	10	399.4 ± 35.8	3.19 ± 0.15	0.21 ± 0.02	0.56 ± 0.02	110.4 ± 29.4	57.6 ± 8.4
DEN + DMH	0.75	7	382.5 ± 13.4	3.38 ± 0.18	0.24 ± 0.02	0.57 ± 0.04	92.9 ± 12.1	54.3 ± 7.43
DEN + DMH	75	7	395.0 ± 21.9	3.35 ± 0.17	0.25 ± 0.04	0.55 ± 0.02	109.4 ± 21.8	62.7 ± 17.8
NSS	-	-	445.0 ± 37.4	3.40 ± 0.11	0.20 ± 0.02	0.55 ± 0.02	75.5 ± 15.6	39.2 ± 8.3
NSS	75	10	428.0 ± 31.7	3.40 ± 0.31	0.19 ± 0.02	0.56 ± 0.03	84.6 ± 20.9	43.0 ± 8.4

* Significantly different from negative control group, *p* < 0.05. ALT: alanine aminotransferase, AST: aspartate aminotransferase, DEN: diethylnitrosamine, DMH: 1,2-dimethylhydrazine, NSS: normal saline solution, VA: vanillic acid.

**Table 2:** The effect of VA on the number of the GST-P positive foci and ACF in DEN- and DMH- initiated rats.

Treatment		Week of Administration	Preneoplastic Lesion			
Carcinogen/NSS	VA (mg kg^−1^ BW)		Liver		Colon	
			No. of GST-P^+^ Foci/cm^2^	Area (mm^2^/cm^2^)	Aberrant Crypt/Rat	Aberrant Crypt/Focus
DEN + DMH	-	-	21.65 ± 8.07 *	1.52 ± 0.72 *	159.33 ± 31.51 *	4.69 ± 0.75 *
DEN + DMH	0.75	10	21.28 ± 6.73	1.58 ± 0.77	163.50 ± 29.40	4.03 ± 0.80
DEN + DMH	75	10	10.15 ± 4.60 **	0.59 ± 0.29 **	138.88 ± 14.93	3.87 ± 0.46
DEN + DMH	0.75	7	22.21 ± 7.47	1.62 ± 0.65	133.00 ± 25.69	4.31 ± 0.49
DEN + DMH	75	7	19.43 ± 4.50	1.36 ± 0.41	151.88 ± 14.80	4.12 ± 0.44
NSS	-		0.00 ± 0.00	0.00 ± 0.00	0.00 ± 0.00	0.00 ± 0.00
NSS	75	10	0.00 ± 0.00	0.00 ± 0.00	0.00 ± 0.00	0.00 ± 0.00

Values expressed as mean ± SD; * Significantly different from negative control group, *p* < 0.05; ** Significantly different from positive control group, *p* < 0.05; ACF: aberrant crypt foci, GST-P: glutathione *S*-transferase placental form, DEN: diethylnitrosamine, DMH: 1,2-dimethylhydrazine, NSS: normal saline solution, VA: vanillic acid.

**Table 3:** Primer sequences for real-time polymerase chain reaction.

Gene	5′-3′ Primer Sequence	References
*Bax*	Forward: 5′-GTT GCC CTC TTC TAC TTT GC-3′ Reverse: 5′-ATG GTC ACT GTC TGC CAT G-3′	[30]
*Caspase-3*	Forward: 5′-CTG GAC TGC GGT ATT GAG AC-3′ Reverse: 5′-CCG GGT GCG GTA GAG TAA GC-3′	[30]
*Cyclin D1*	Forward: 5′-GTC GAG AAG AGA AAG CTC TG-3′ Reverse: 5′-TTA AAA GCC TCC TGT GTG AA-3′	[31]
*Nrf-2*	Forward: 5′-GCC AGC TGA ACT CCT TAG AC-3′ Reverse: 5′-GAT TCG TGC ACA GCA GCA-3′	[32]
*GSTA-5*	Forward: 5′-ACC CCT TTC CCT CTG CTG AA-3′ Reverse: 5′-AAA CAT CAG AGC CTG GAT TAC AAG-3′	NM_001010921.1
*CYP2E1*	Forward: 5′-CCT TTC CCT CTT CCC ATC C-3′ Reverse: 5′-AAC CTC CGC ACA TCC TTC C-3′	[33]
*Bad*	Forward: 5′-GGA GCA TCG TTC AGC AGC AG-3′ Reverse: 5′-CCA TCC CTT CAT CTT CCT CAG TC-3′	[34]
*Bcl-2*	Forward: 5′-CTG GTG GAC AAC ATC GCT CTG-3′ Reverse: 5′-GGT CTG CTG ACC TCA CTT GTG-3′	[35]
*β-Actin*	Forward: 5′-ACA GGA TGC AGA AGG AGA TTA C-3′ Reverse: 5′-AGA GTG AGG CCA GGA TAG A-3′	[36]

## Data Availability

The data presented in this study are available on request from the corresponding author.

## Sample Availability

Samples of the compounds are available from the authors.
